# The Impact of Tumor Cell-Intrinsic Expression of Cyclic GMP-AMP Synthase (cGAS)-Stimulator of Interferon Genes (STING) on the Infiltration of CD8^+^ T Cells and Clinical Outcomes in Mismatch Repair Proficient/Microsatellite Stable Colorectal Cancer

**DOI:** 10.3390/cancers15102826

**Published:** 2023-05-18

**Authors:** Shotaro Nakajima, Akinao Kaneta, Hirokazu Okayama, Katsuharu Saito, Tomohiro Kikuchi, Eisei Endo, Takuro Matsumoto, Satoshi Fukai, Mei Sakuma, Takahiro Sato, Kosaku Mimura, Motonobu Saito, Zenichiro Saze, Wataru Sakamoto, Hisashi Onozawa, Tomoyuki Momma, Koji Kono

**Affiliations:** 1 Department of Multidisciplinary Treatment of Cancer and Regional Medical Support, Fukushima Medical University School of Medicine, Fukushima 960-1295, Japan; 2 Department of Gastrointestinal Tract Surgery, Fukushima Medical University School of Medicine, Fukushima 960-1295, Japan; 3 Department of Blood Transfusion and Transplantation Immunology, Fukushima Medical University School of Medicine, Fukushima 960-1295, Japan

**Keywords:** colorectal cancer (CRC), pMMR/MSS CRC, tumor cell-intrinsic cGAS-STING, CD8^+^,CD4^+^ T cells

## Abstract

**Simple Summary:**

Although the tumor cell-intrinsic cyclic GMP-AMP synthase (cGAS)-stimulator of interferon genes (STING) pathway plays a crucial role in activating immune cells in the tumor microenvironment in colorectal cancer (CRC), its impact on the infiltration of immune cells and clinical outcomes in patients with mismatch repair proficient/microsatellite stable (pMMR/MSS) CRC has not been thoroughly investigated. In this study, we examine the expression pattern of cGAS-STING in tumor cells and its effect on the infiltrations of CD8^+^ and CD4^+^ T cells, as well as clinical outcomes including survival and recurrence in patients with pMMR/MSS CRC. Our current findings may offer novel insights and therapeutic strategies for patients with pMMR/MSS CRC.

**Abstract:**

The cyclic GMP-AMP synthase (cGAS)-stimulator of interferon genes (STING) pathway plays a crucial role in activating immune cells in the tumor microenvironment, thereby contributing to a more favorable response to immune checkpoint inhibitors (ICI) in colorectal cancer (CRC). However, the impact of the expression of cGAS-STING in tumor cells on the infiltration of CD8^+^ T cells and clinical outcomes in mismatch repair proficient/microsatellite stable (pMMR/MSS) CRC remains largely unknown. Our findings reveal that 56.8% of all pMMR CRC cases were cGAS-negative/STING-negative expressions (cGAS^−^/STING^−^) in tumor cells, whereas only 9.9% of all pMMR CRC showed cGAS-positive/STING-positive expression (cGAS^+^/STING^+^) in tumor cells. The frequency of cGAS^+^/STING^+^ cases was reduced in the advanced stages of pMMR/MSS CRC, and histone methylation might be involved in the down-regulation of STING expression in tumor cells. Since the expression level of cGAS-STING in tumor cells has been associated with the infiltration of CD8^+^ and/or CD4^+^ T cells and the frequency of recurrence in pMMR/MSS CRC, decreased expression of cGAS-STING in tumor cells might lead to poor immune cell infiltration and worse prognosis in most pMMR/MSS CRC patients. Our current findings provide a novel insight for the treatment of patients with pMMR/MSS CRC.

## 1. Introduction

Colorectal cancer (CRC) is the third most common type of cancer and the second leading cause of cancer-related deaths worldwide [1]. CRC is categorized into two major subtypes, namely mismatch repair proficient/microsatellite stable (pMMR/MSS) tumors and mismatch repair deficient/microsatellite instability-high (dMMR/MSI-H) tumors [2,3]. About 15% of all CRC cases display MSI-H phenotype, which is caused by the germline mutations of MMR genes, including *MutL homolog 1* (*MLH1*), *MutS homolog 2* (*MSH2*), *MutS homolog 6* (*MSH6*)*,* and *PMS1 homolog 2* (*PMS2*) in Lynch syndrome cases (1–3%) [4,5,6,7,8,9] or somatic hypermethylation of CpG islands surrounding the promoter region of *MLH1* in sporadic MSI-H cases (3–15%) [10,11]. The remaining 85% of all CRC cases are pMMR/MSS, most of which exhibit the chromosomal instability (CIN) phenotype [12]. Compared to pMMR/MSS CRCs, dMMR/MSI-H CRCs are known to exhibit a high degree of immune cell infiltration, particularly CD8^+^ T cells, due to the high levels of tumor mutational burden and neoantigen load [13,14]. As a result, the use of immune checkpoint inhibitors (ICI) can lead to a sustained response and significant improvement in patient survival with dMMR/MSI-H CRC [15]. However, current results regarding ICI therapy in patients with pMMR/MSS CRC are disappointing.

The cyclic GMP-AMP synthase (cGAS)-stimulator of interferon genes (STING) pathway has a pivotal role for the activation of cytosolic DNA-mediated type I interferon (IFN) response [16,17]. Upon recognition of cytosolic double-stranded DNA, cGAS generates cyclic GMP-AMP (cGAMP), which functions as a second messenger to activate STING and its downstream transcription factors such as interferon regulatory factor 3 and nuclear factor-κB, resulting in the production of type I IFN and inflammatory cytokines/chemokines, including C-X-C motif ligand (CXCL) 9/10/11 and C-C chemokine ligand-5 (CCL5) [18,19]. Substantial amounts of cytoplasmic dsDNA are found under pathological conditions, including cancer [20], and it has been reported that cytoplasmic dsDNA in cancer cells may contribute to type I IFN-dependent priming of tumor cell-specific T cell immunity [21]. Several previous studies have established the significance of the cGAS-STING pathway in tumor cells for activating immune cells in the tumor microenvironment (TME) in CRC [22,23]. Our recent findings indicated that the expression of tumor cell-intrinsic cGAS-STING remained elevated in dMMR/MSI-H CRC, promoting increased infiltration of CD8^+^ T cells [24]. In contrast, pMMR/MSS exhibited low levels of tumor cell-intrinsic expression of cGAS-STING, resulting in reduced infiltration of CD8^+^ T cells [24]. Of importance is that our previous report suggested that, even in pMMR/MSS CRC, a small subset of the patients (approximately 10% of all pMMR/MSS cases) displayed high infiltration of CD8^+^ T cells and activation of IFN response [25], which could contribute to a favorable response to ICI treatment [15]. However, the expression pattern of cGAS-STING in tumor cells and its association with the infiltration of CD8^+^ T cells, as well as their impact on clinical outcomes, in pMMR/MSS CRC are largely unexplored.

In this study, we examine the expression level of tumor cell-intrinsic cGAS-STING and its association with the infiltration of CD8^+^ T cells and clinical outcomes in pMMR/MSS CRC by analyzing immunohistochemistry (IHC) of our cohort and gene expression datasets obtained from The Cancer Genome Atlas (TCGA) and the Gene Expression Omnibus (GEO).

## 2. Materials and Methods

### 2.1. Patients and Specimens

We recruited 283 patients with primary CRC (FMU cohort, pMMR; *n* = 243, dMMR; *n* = 40) (Table 1 and Table S1) who underwent surgical resection between 2002 and 2013 at the Department of Gastrointestinal Tract Surgery of Fukushima Medical University Hospital (Fukushima, Japan). The clinical and pathological data were retrospectively collected from medical records. This study was approved by the Institutional Ethical Committee of Fukushima Medical University, and all procedures were conducted in accordance with the Helsinki Declaration.

### 2.2. IHC

Paraffin-embedded 4-μm CRC tissue sections, which were fixed in 10% formaldehyde, underwent processing using a standard histological protocol. Briefly, the tissue sections were deparaffinized in xylene, rehydrated using a series of ethanol, and treated with 0.3% hydrogen peroxide in methanol to block Endogenous peroxidases. Following antigen retrievals by autoclave using Target Retrieval Solution pH6.0 or pH9.0 (Dako/Agilent Technologies, Santa Clara, CA, USA), the sections were incubated overnight at 4℃ with the following primary antibodies: anti-cGAS mAb (#79978; dilution 1:200), anti-STING mAb (#13647; dilution 1:200), anti-programmed death-ligand 1 (PD-L1) mAb (#13684; dilution 1:400) (Cell Signaling Technology, Danvers, MA, USA), anti-CD4 mAb (M7310; dilution 1:100), anti-CD8 mAb (M7103; dilution 1:200) (Dako/Agilent Technologies), and anti-Foxp3 mAb (ab20034; dilution 1:200) (Abcam, Cambridge, UK). After primary antibody incubation, the sections were washed and incubated with horseradish peroxidase-conjugated secondary antibodies (K4003 or K4001; Dako/Agilent Technologies). Detection of peroxidase was performed using diaminobenzidine peroxidase substrate (Dojindo Molecular Technology, Kumamoto, Japan), and nuclei were counterstained with Mayer Hematoxylin Solution (FUJIFILM Wako Pure Chemical Corporation, Osaka, Japan).

To evaluate the expression of cGAS and STING in tumor cells, whole tissue sections stained with anti-cGAS or anti-STING antibodies were captured using Nanozoomer Digital Pathology slide scanner (Hamamatsu Photonics, Hamamatsu, Japan). The staining intensity in the cytoplasm was graded as follows: 0 (none), 1+ (weak), 2+ (moderate), or 3+ (strong) (Figure S1A). Regarding the extent score, the scanned whole tissue image was partitioned into multiple regions and the proportion of stained cytoplasm was determined by calculating the mean value of each region. The extent score was estimated based on the percentage of the area of stained cytoplasm (0 for no staining at all, 1 for <10%, 2 for 10–50%, and 3 for >50% of tumor cells stained). The final score was determined by multiplying the extent score and intensity score (IHC signal, 0–9), and IHC signal ≧3 was considered as positive expression (Figure S1B) [22]. For PD-L1 staining, only membranous staining without any cytoplasmic staining was evaluated. Tumor specimens were classified as PD-L1-positive when more than 1% of the tumor cells displayed membranous staining of any intensity, as previously described [26]. To assess CD4^+^, CD8^+^, and Foxp3^+^ TILs, the invasive front region of the tumor was reviewed in four independent areas, and the number of lymphocytes was counted at a magnification of ×400, as previously described [25]. Two observers (S.N. and A.K.) evaluated the IHC analyses without access to any clinical and pathological records. In cases of contradictory scores between the two observers, samples were reevaluated jointly until a consensus was reached.

To determine the MMR status, CRC tissue sections were stained with primary antibodies for MMR proteins (MLH1: M3640, dilution 1:50; MSH2: M3639, dilution 1:50; MSH6: M3646, dilution 1:200; PMS2: M3647, dilution 1:50) (Dako/Agilent Technologies) as previously described [27]. Loss of at least one MMR protein was defined as dMMR, while tumors with intact MMR protein expression were defined as pMMR.

### 2.3. Data Analyses of TCGA and GEO Database

We obtained publicly accessible datasets of mRNA expression of genes for pMMR/MSS colon and rectal adenocarcinoma (including MSI-low cases) from cBioPortal (http://www.cbioportal.org/) [28] accessed on 8 November 2022 and the GEO database accessed on 16 November 2022. The log_2_ signal intensity was obtained from TCGA (COADREAD) (MSI-low/MSS cases; *n* = 450) and GSE39582 (pMMR cases; *n* = 444), and we calculated multi-gene expression signatures, including CD8 effector genes (*GZMK*, *CD3E*, *CD3G*, *CXCR3*, *CD3D*, *BCL11B*, *CD28*, *KLRG1*, *IL7R*) and CD4 mature genes (*IGFBP4*, *ITM2A*, *AMIGO2*, *TRAT1*, *CD40LG*, *ICOS*, *RCAN3*) [29]. The median values were utilized to classify pMMR/MSS CRCs as cGAS-high or cGAS-low, STING-high or STING-low, CXCL9/10/11-high or CXCL9/10/11-low, and CCL5-high or CCL5-low.

### 2.4. Statistical Analysis

Statistical analyses were conducted utilizing Graph pad Prism Version 9.3.0 (Graph Pad Software, San Diego, CA, USA). To ascertain differences between two variables, unpaired *t* tests or Mann–Whitney tests were used. For multigroup comparisons, Chi-square tests, Kruskal–Wallis tests with post hoc Dunn tests or uncorrected Dunn’s tests were used. Kaplan–Meier estimates calculate the probability of 10-year overall survival (OS) and 5-year recurrence-free survival (RFS), and the difference between the two groups was analyzed by the log-rank test. Statistical significance was considered at *p*-values less than 0.05.

## 3. Results

### 3.1. The Expression Patterns of Tumor Cell-Intrinsic cGAS-STING in pMMR/MSS CRC

**Figure 1:**
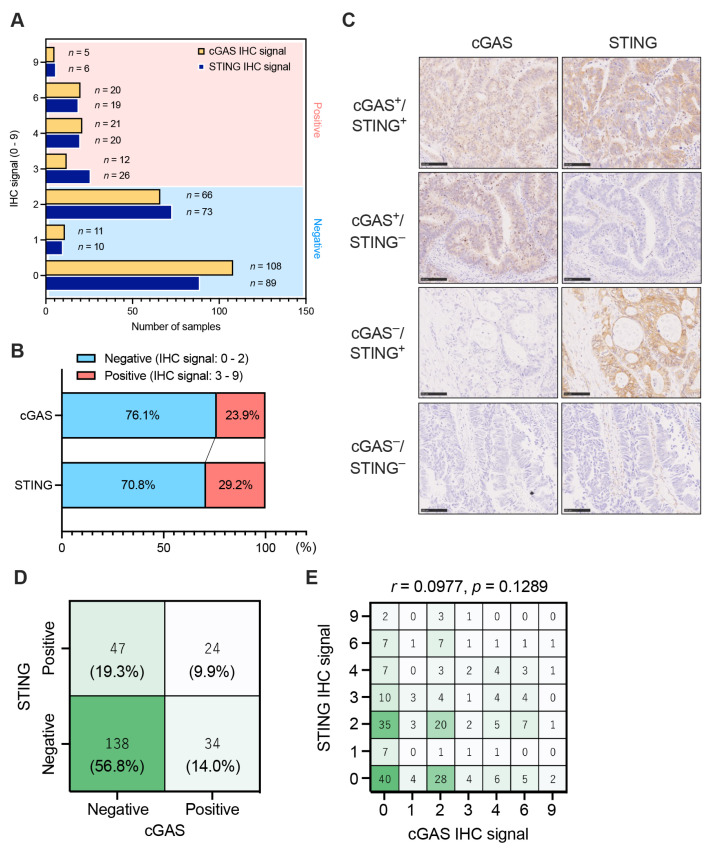
The expression pattern of cGAS-STING in tumor cells in pMMR CRC. (**A**) Distribution of IHC signals for cGAS-STING in pMMR CRC. cGAS- or STING-positive cases are highlighted in red, and cGAS- or STING-negative are highlighted in blue. (**B**) The percentage of positive or negative cases for the expression of cGAS or STING in tumor cells in pMMR CRC. cGAS- or STING-positive cases are highlighted in red, and cGAS- or STING-negative are highlighted in blue. The percentages of cGAS-STING positivity are indicated in the graph. (**C**) Representative IHC staining images for cGAS and STING in cGAS-positive/STING-positive (cGAS^+^/STING^+^), cGAS-positive/STING-negative (cGAS^+^/STING^−^), cGAS-negative/STING-positive (cGAS^−^/STING^+^), and cGAS-negative/STING-negative (cGAS^−^/STING^−^) pMMR CRCs. Scale bars, 100 μm. (**D**) Proportion of cGAS^+^/STING^+^, cGAS^+^/STING^−^, cGAS^−^/STING^+^, and cGAS^−^/STING^−^ pMMR CRCs. (**E**) Correlations between the expression of cGAS-STING (IHC signals) in tumor cells in pMMR CRC. The intensities of the heatmap represent number of each proportion, with values ranging from 0 to 138 (**D**) or from 0 to 40 (**E**) shown as white and green. Statistical significance was determined by the Spearman correlation test (**E**).

We first evaluated the expression level of cGAS-STING in tumor cells in pMMR CRC (*n* = 243) by assessing the IHC signal (ranging from 0 to 9), which was determined by multiplying the extent score (ranging from 0 to 3: 0 for no staining signal at all, 1 for <10%, 2 for 10–50%, and 3 for >50% of tumor cells stained) and the intensity score (0–3+: 0—none, 1+—weak, 2+—moderate, or 3+—strong). A value of IHC signal ≧3 was considered as the presence of the expression of cGAS and STING in tumor cells in CRC (Figure S1) [22]. The IHC analyses revealed that 76.1% of all pMMR CRCs were cGAS-negative expressions, and 70.8% of all pMMR CRCs were STING-negative expressions in tumor cells (Figure 1A,B). We observed four distinct expression patterns of cGAS-STING in CRC tumor cells, which included cases that expressed both cGAS and STING (cGAS^+^/STING^+^), those that expressed cGAS only (cGAS^+^/STING^−^), those that expressed STING only (cGAS^−^/STING^+^), and those that lacked expressions of both cGAS and STING (cGAS^−^/STING^−^) (Figure 1C). Representative IHC staining of the whole tissue sections were shown in Figure S2. Remarkably, more than half of the pMMR CRC cases showed cGAS^−^/STING^−^, whereas only a small fraction of pMMR CRC cases (9.9%) were cGAS^+^/STING^+^ (Figure 1D). In contrast, in dMMR CRC, cGAS^−^/STING^−^ CRC cases were observed in only 20% of all dMMR CRCs (Figure S3A), and 52.5% of dMMR CRC lacked cGAS expression, while 40.0% lacked STING expression in tumor cells (Figure S3B). Furthermore, our result revealed no significant correlation between the expression of cGAS and STING in tumor cells in pMMR CRC (*r* = 0.0977, *p* = 0.1289) (Figure 1E), suggesting that the expression of these molecules in tumor cells may be regulated by distinct mechanisms in pMMR CRC. Consistent with this result, the analyses of TCGA and GSE39582 datasets also demonstrated no significant associations between mRNA expression of cGAS and STING in pMMR/MSS CRC (Figure S4A,B).

### 3.2. Down-Regulation of the Expression of cGAS-STING in Tumor Cells in the Advanced Stages of pMMR CRC

**Figure 2:**
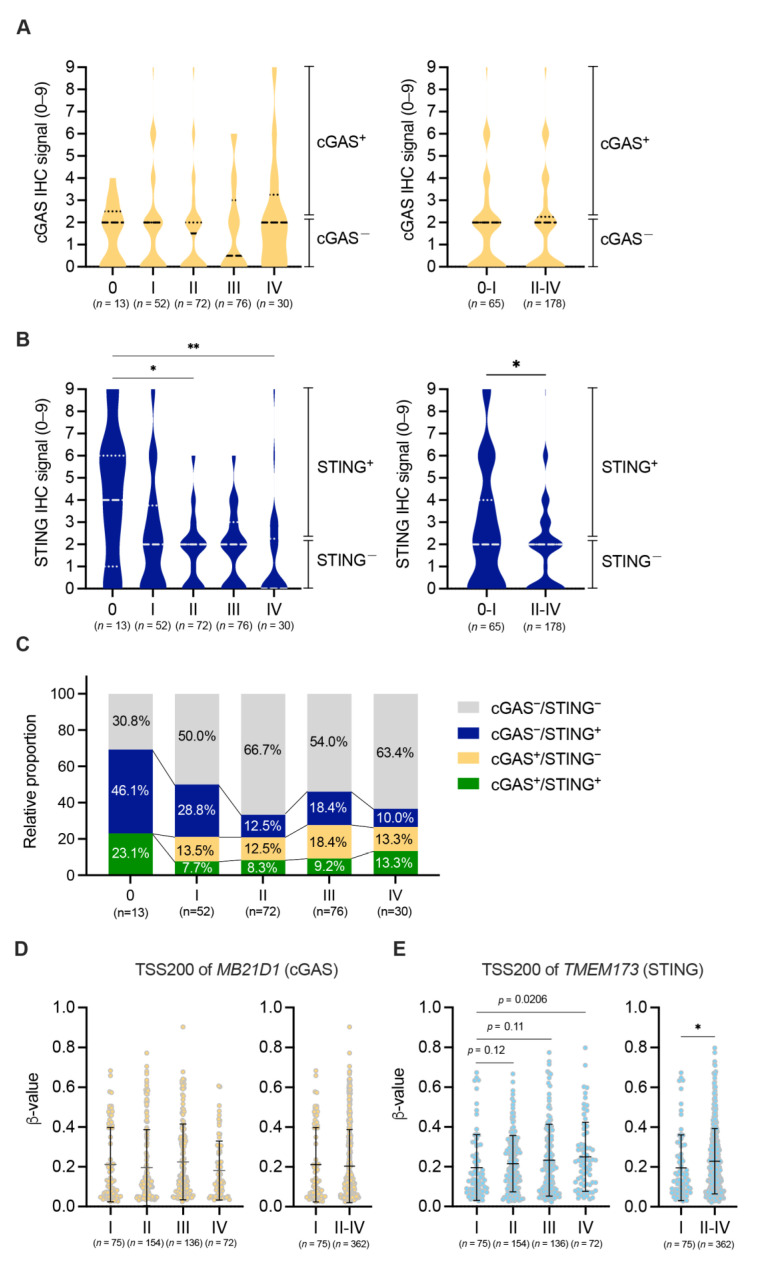
Decreased expression of cGAS-STING in tumor cells in the advanced stages of pMMR/MSS CRC. (**A**,**B**) Correlations between IHC signals of cGAS (**A**) or STING (**B**) and TNM stage (**left**) or early–late stages (**right**) in patients with pMMR CRC (FMU cohort). Lines in the right side of the graphs indicate the ranges of cGAS-STING-positive or cGAS-STING-negative. (**C**) Correlations between the percentages of cGAS^+^/STING^+^ (green), cGAS^+^/STING^−^ (yellow), cGAS-/STING^+^ (blue), or cGAS^−^/STING^−^ (grey) and TNM stage in patients with pMMR CRC (FMU cohort). (**D**,**E**) Correlations between histone methylation levels in the promoter region of cGAS (**D**) or STING (**E**) and TNM stage (**left**) or early–late stages (**right**) in patients with MSS CRC (TCGA cohort). Medians and quartiles are shown in violin plots. Means ± SD are shown in dot plots. * *p* < 0.05, ** *p* < 0.01. Statistical significance was determined by the Mann–Whitney test ((**A**,**B**,**D**,**E**)**-**(**right**)), Kruskal–Wallis test with post hoc Dunn’s multiple comparisons test ((**A**,**B**)**-**(**left**)) or uncorrected Dunn’s test ((**D**,**E**)**-**(**left**)).

We next examined the association between the expression of cGAS-STING in tumor cells and clinico-pathological characteristics of patients with pMMR CRC. The expression of cGAS was significantly higher in tumor cells of proximal colon than tumor cells of distal and rectum colons (Figure S5A,B). In addition, we observed that poorly differentiated tumor cells exhibited higher levels of cGAS expression in contrast to well-differentiated tumor cells in pMMR CRCs (Figure S5C,D). A significant association was observed between the location of tumor and the proportion of cGAS/STING positivity in pMMR/MSS CRC (*p* < 0.05) (Table 1). Interestingly, we found that tumor cell-intrinsic expression of STING, but not cGAS, was decreased in the advanced stages of pMMR CRC (Figure 2A,B and Figure S6), and the percentages of cases lacking cGAS-STING expression in tumor cells increased in stages II and IV of pMMR CRC (Figure 2C). A significant inverse correlation was found between T stage and the proportion of cGAS/STING positivity in pMMR/MSS CRC (*p* < 0.05) (Table 1). Konno et al. previously demonstrated that histone methylation in promoter regions of cGAS and STING were significantly enhanced in several types of cancer, including CRC [30]. Indeed, by analyzing the datasets from TCGA, we observed that the histone methylation level in the promoter region of STING, but not cGAS, was significantly increased in the advanced stages of MSS CRC (Figure 2D,E). These results suggest that the expression of STING in tumor cells was down-regulated in the advanced stages of pMMR/MSS CRC, which might depend on the increased histone methylation level in the promoter region of STING.

### 3.3. Associations between the Tumor Cell-Intrinsic Expression of cGAS-STING and the Infiltration of CD8^+^ and CD4^+^ T Cells in pMMR/MSS CRC

**Figure 3:**
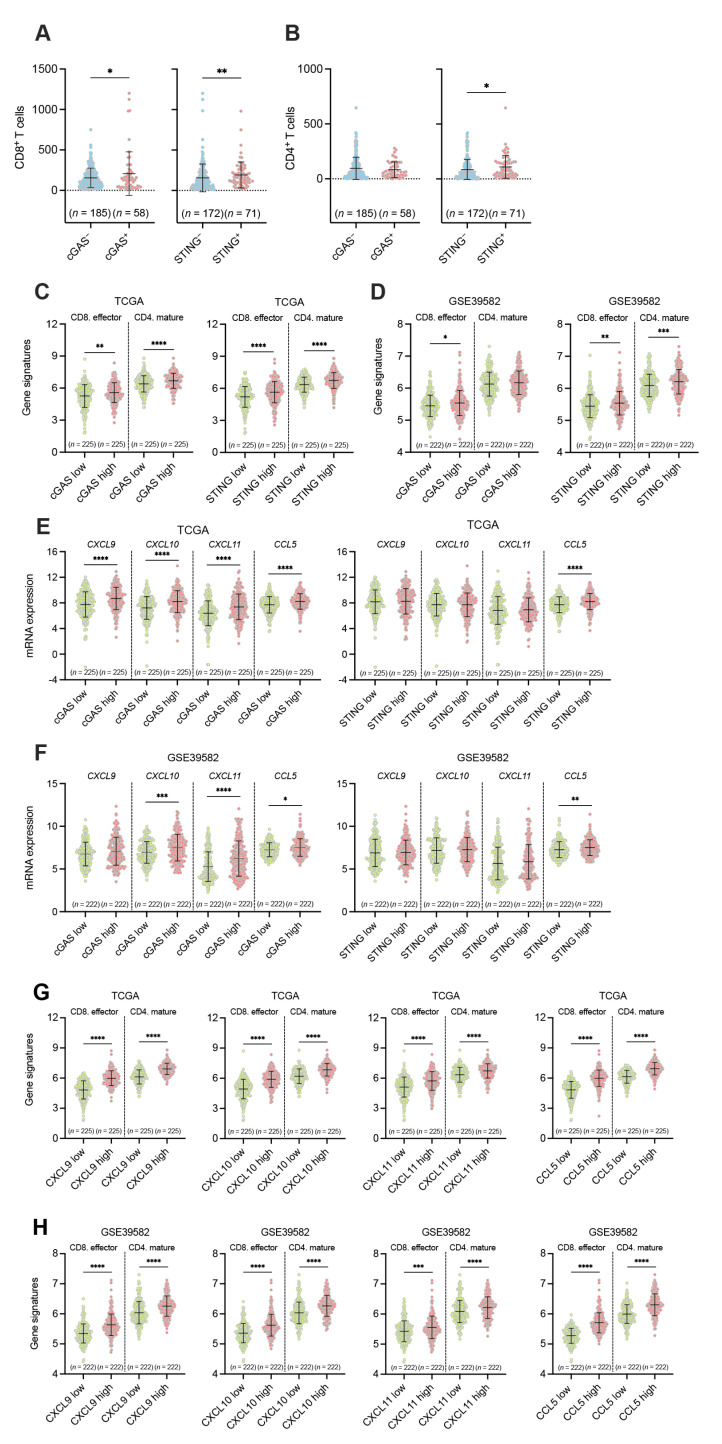
Involvement of the tumor cell-intrinsic expression of cGAS-STING in the infiltration of CD8^+^ and CD4^+^ T cells in pMMR CRC. (**A**,**B**) The number of tumor-infiltrating CD8^+^ T cells (**A**) or CD4^+^ T cells (**B**) between cGAS^+^ and cGAS^−^, or STING^+^ and STING^−^ pMMR CRCs (FMU cohort). (**C**,**D**) The expression of CD8 effector gene signature or CD4 mature gene signature between cGAS-high and cGAS-low (**left**), or STING-high and STING-low (**right**) MSS CRC (TCGA cohort) (**C**) or in pMMR CRC (GSE39582 cohort) (**D**). (**E**,**F**) The expression of *CXCL9/10/11* and *CCL5* between cGAS-high and cGAS-low (**left**), or STING-high and STING-low (**right**) MSS CRC (TCGA cohort) (**E**) or pMMR CRC (GSE39582 cohort) (**F**). (**G**,**H**) The expression of CD8 effector or CD4 mature gene signatures between CXCL9/10/11^-^high and CXCL9/10/11-low, or CCL- high and CCL5-low MSS CRC (TCGA cohort) (**G**) or pMMR CRC (GSE39582 cohort) (**H**). The median values were utilized to classify cGAS-high or cGAS-low, STING-high or STING-low, CXCL9/10/11-high or CXCL9/10/11-low, and CCL5-high or CCL5-low pMMR/MSS CRCs. Means ± SD are shown in dot plots. Low groups indicate blue dots or green dots, and high groups indicate red dots. * *p* < 0.05, ** *p* < 0.01, *** *p* < 0.001, **** *p* < 0.0001. Statistical significance was determined by the unpaired *t* test ((**A**,**B**)**-**(**left**)) or Mann–Whitney test (**others**).

We also examined the association between the expression of cGAS-STING in tumor cells and the infiltrations of CD8^+^ T cells and CD4^+^ T cells in pMMR CRC. The number of CD8^+^ T cells and CD4^+^ T cells was significantly higher in STING^+^ pMMR CRCs compared to STING^−^ pMMR CRCs, whereas cGAS expression was positively associated with only CD8^+^ T cells, but not CD4^+^ T cells, in pMMR CRCs (Figure 3A,B). In addition, we analyzed datasets obtained from TCGA and GSE39582 to examine the expression of CD8 effector and CD4 mature gene signatures between cGAS-high and cGAS-low or STING-high and STING-low pMMR/MSS CRCs. Upon dividing pMMR/MSS CRC cases into cGAS-high or cGAS-low and STING-high or STING-low groups, the expression of CD8 effector and CD4 mature gene signatures was markedly higher in cGAS-high and STING-high pMMR/MSS CRCs than in cGAS-low and STING-low pMMR/MSS CRCs, respectively (Figure 3C,D), suggesting that the infiltration of CD8^+^ effector T cells and CD4^+^ mature T cells might be associated with the expression of cGAS and STING in pMMR/MSS CRCs.

We also examined the association between the expression of cGAS-STING in tumor cells and the infiltrations of Treg cells (Foxp3^+^ cells) in pMMR CRC by IHC. We found that there was no association between cGAS-STING expression in tumor cells and the infiltration of Treg cells in pMMR CRC, suggesting that the CD4^+^ population infiltrated through tumor cell-intrinsic expressions of STING in pMMR CRC might not be Treg cells (Figure S7).

It has been reported that CXCL9/10/11 and CCL5 have been recognized as cGAS-STING-dependent genes, and they function as chemo-attractants for CD8^+^ T cells and CD4^+^ T cells in the TME [31]. Our analyses revealed that these chemokines were also highly expressed in cGAS-high or STING-high pMMR/MSS CRCs than cGAS-low or STING-low pMMR/MSS CRCs (Figure 3E,F). Moreover, the expression of CD8 effector and CD4 mature genes was significantly and positively associated with the gene expression of *CXCL9*, *CXCL10*, *CXCL11*, and *CCL5* in pMMR/MSS CRC, suggesting that the production of CXCL9/10/11 and CCL5 might contribute to the infiltration of CD8^+^ T cells and CD4^+^ T cells in pMMR/MSS CRC (Figure 3G,H). Taken together, based on the results of our IHC analyses, decreased expression of STING in tumor cells might be involved in the low infiltration of both CD8^+^ and CD4^+^ T cells, and low expression of cGAS in tumor cells might be involved in the low infiltration of CD8^+^ T cells in pMMR/MSS CRC.

### 3.4. Associations between the Tumor Cell-Intrinsic Expression of cGAS-STING and Clinical Outcomes in Patients with pMMR/MSS CRC

**Figure 4:**
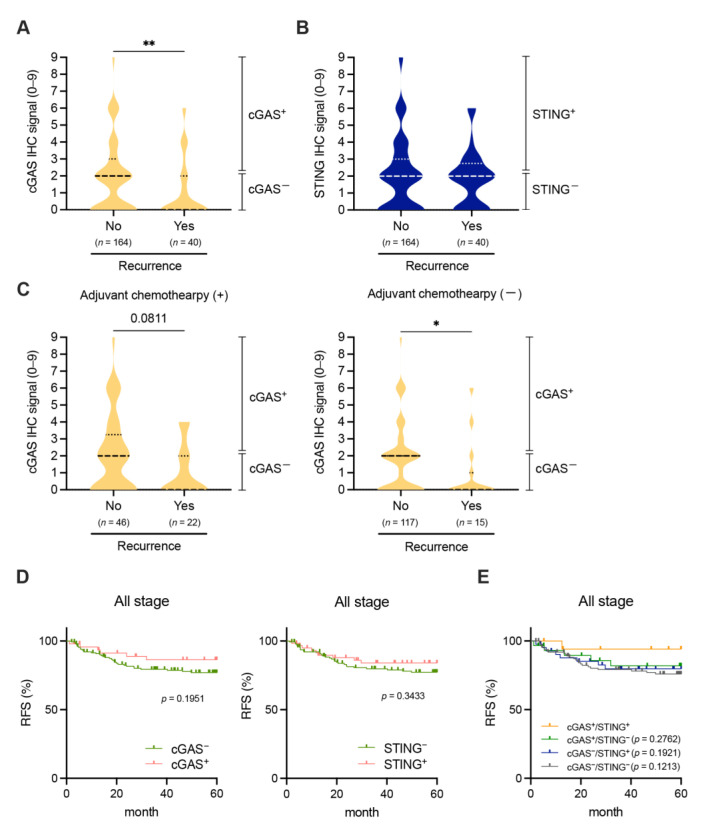
Involvement of the tumor cell-intrinsic expression of cGAS-STING in the frequency of recurrence in patients with pMMR CRC. (**A**,**B**) Correlations between IHC signals of cGAS (**A**) or STING (**B**) and 5-year recurrence in patients with pMMR CRC. (**C**) Correlations between IHC signals of cGAS and 5-year recurrence with or without adjuvant chemotherapy in patients with pMMR CRC. (**D**) Kaplan–Meier curves for 5-year RFS in patients with cGAS^+^ (red) or cGAS^−^ (green) (**left**) and STING^+^ (red) or STING^−^ (green) (**right**) pMMR CRCs. (**E**) Kaplan–Meier curves for 5-year RFS in patients with cGAS^+^/STING^+^ (yellow), cGAS^+^/STING^−^ (green), cGAS^-^/STING^+^ (blue), and cGAS^−^/STING^−^ (grey) pMMR CRCs. *p*-values vs. cGAS^+^/STING^+^ pMMR CRC are shown in the graph. Medians and quartiles are shown in violin plots. * *p* < 0.05, ** *p* < 0.01. Statistical significance was determined by the Mann–Whitney test (**A**–**C**) or log-rank test (**D**,**E**).

We finally examined the association between the expression of cGAS-STING in tumor cells and clinical outcomes in patients with pMMR/MSS CRC. The expression of cGAS-STING in tumor cells did not have a significant impact on patient survival with pMMR CRC within ten years after curative resection (Figure S8A,B). However, tumor cell-intrinsic expression of cGAS, but not STING, was significantly reduced in pMMR/MSS CRC from patients who experienced recurrence within five years after curative resection, regardless of whether they had received adjuvant chemotherapy (Figure 4A–C). Moreover, Kaplan–Meier analyses revealed that the tumor cell-intrinsic expression of cGAS-STING tended to be positively associated with 5-year recurrence-free survival (RFS), but not with 10-year overall survival (OS), in patients with pMMR CRC, although these findings were not significant (Figure 4D and Figure S8C). We also performed Kaplan–Meier analyses for 10-year OS for each stage. However, the expression of cGAS-STING in tumor cells did not have a significant impact on patient OS in every stage (Figure S9). When comparing 5-year RFS and 10-year OS among patients with cGAS^+^/STING^+^, cGAS^+^/STING^−-^, cGAS^−^/STING^+^, and cGAS^−^/STING^−^ pMMR CRCs, patients with cGAS^+^/STING^+^ pMMR CRCs exhibited a tendency to have better 5-year RFS, but not 10-year OS, compared to the other three groups of patients with pMMR CRC (Figure 4E and Figure S8D).

## 4. Discussion

In this study, for the first time, we revealed that approximately 60% of all pMMR CRC cases were found to be cGAS^−^/STING^−^ (Figure 1), whereas the proportion of cGAS^+^/STING^+^ cases in pMMR CRC was merely 9.9% (Figure 1). The proportion of cGAS^+^/STING^+^ cases decreased in the advanced stages of pMMR/MSS CRC (Figure 2). Since the methylation level in the promoter region of STING was enhanced in the advanced stages of MSS CRC (Figure 2), it is possible that epigenetic changes might be implicated in the down-regulation of STING in tumor cells. We observed a poor infiltration of CD8^+^ and CD4^+^ T cells and an increased frequency of recurrence in pMMR/MSS CRC with the reduced expression or lost expression of cGAS-STING in tumor cells (Figure 3 and Figure 4).

The recent spatial multi-omic profiling conducted by Heide et al. investigated the genomic and epigenomic changes in CRC. Their findings suggested that alterations of somatic chromatin accessibility in genes, especially those which that regulate interferon response, are associated with enhanced tumorigenesis in CRC [32]. Furthermore, Konno et al. reported a significant and dramatic increase in histone methylation levels in the promoter region of cGAS and STING in tumor tissues, compared to adjacent normal tissues, in several types of cancers including CRC [30]. In the present study, we also find that, by analyzing the datasets from TCGA, the methylation level in the promoter region of STING significantly increased in the advanced stages of pMMR/MSS CRC (Figure 2). Therefore, the epigenetic alteration, particularly histone methylation in the promoter region of STING, might be one of the critical factors that lead to a down-regulation of STING expression in tumor cells, resulting in less activation and infiltration of immune cells, including CD8^+^ T cells, in pMMR/MSS CRC.

No correlation was observed between cGAS and STING expression in tumor cells in pMMR/MSS CRC as determined through IHC analysis of our cohort and the gene expression analyses of public datasets from TCGA and GEO, suggesting that different regulatory mechanisms for the expression of cGAS and STING in tumor cells might exist in pMMR/MSS CRCs. Notably, the histone methylation level in the promoter region of STING was decreased in the advanced stages of pMMR/MSS CRCs, while the histone methylation level in the promoter region of cGAS did not differ across all pathological stages (Figure 2D,E). Interestingly, Xia et al. demonstrated that histone deacetylase inhibitors (HDACi) could rescue cGAS expression in some colon adenocarcinoma cells [22], indicating that histone acetylation of cGAS gene is involved in the suppression of its expression in colorectal tumor cells. Based on our current findings and previous reports, the expression of cGAS and STING in tumor cells might be regulated through different epigenetic alterations, such as histone methylation and acetylation, in pMMR/MSS CRC.

Most of the pMMR/MSS CRCs arise via the conventional adenoma–carcinoma sequence, which involves the activation of oncogenes such as *KRAS* and the inactivation of tumor suppressors, including *APC*, *SMAD4*, and *TP53* [33,34,35,36]. During this process, epigenetic modification, such as histone methylation of tumor suppressor genes and particularly STING, might be induced at the advanced phases of tumorigenesis, resulting in further enhancement of tumor growth and metastasis. On the other hand, most dMMR/MSI-H CRCs are generated through the serrated pathway, characterized by *BRAF* mutation and a CpG island methylation phenotype [37,38]. During this process, hypermethylation might occur in several genes especially MLH1 but not STING, resulting in the generation of MSI-H cancers with high expression of cGAS-STING in tumor cells concomitant with immune cell activation in the TME.

We found that tumor cell-intrinsic expression of cGAS, but not STING, was significantly reduced in pMMR/MSS CRC from patients who experienced recurrence within five years after curative resection. However, the regulatory mechanisms by which cGAS expression suppresses tumor recurrence in patients with CRC remain largely unknown. In a previous study, Yang et al. reported that cGAS is a crucial factor in cellular senescence induced by DNA damage [39]. In response to cytosolic DNA, cGAS entered the nucleus and associated with chromatin DNA to regulate proper cell cycle progression during mitosis in proliferating cells via a STING-independent mechanism. Indeed, cGAS deletion accelerated spontaneous immortalization of cells, which might be linked to several human diseases, including cancer and age-related diseases [39]. Therefore, one possibility is that a similar mechanism might be involved in the increased frequency of recurrence in patients with pMMR/MSS CRC with low expression of cGAS in tumor cells. Further investigation is needed to understand the role of tumor-cell intrinsic cGAS-STING, not only in the activation of immune cells in the TME but also in tumor cell proliferation in CRC.

In a previous study, Lu et al. demonstrated that guadecitabine, a next generation DNA methyltransferase inhibitor (DNMTi), significantly up-regulated the expression of CXCL9/10/11 as well as major histocompatibility class I in breast cancer cells [40]. DNMTi administration was shown to increase tumor-infiltrating CD8^+^ T cells and promote breast tumor regression in mice [40]. Moreover, Li et al. analyzed the effects of 5-azacitidine, a nucleoside analogue of cytidine that specifically inhibits DNMT, on integrative gene expression and methylation analysis of 63 cell lines, including COLO201 and HT29 MSS colorectal cancer cell lines. Treatment of these cancer cells with low doses of DNMTi strongly up-regulated genes related to immunomodulatory pathways, including IFN signaling, cytokines/chemokines, and antigen processing and presentation [41]. They also found that combination therapy with DNMTi and entinostat, an HDAC inhibitor (HDACi), induced gene expression of immunomodulatory pathways in tumor tissues, including colorectal cancer tissues [41]. Importantly, Kuang et al. conducted a phase 2 single-arm trial assessing the efficacy and tolerability of pembrolizumab, a humanized anti-PD-1 IgG4 antibody, and 5-azacitidine in patients with chemotherapy-refractory metastatic CRC (mCRC) [42]. Although the clinical activity of the combination of pembrolizumab and 5-azacytidine was modest in the treatment for chemotherapy-refractory mCRC, the expression of several immune gene sets and the density of CD8^+^ tumor-infiltrating lymphocytes was increased in CRC tissues from patients after the treatment [42]. Tumor-cell intrinsic STING might partially contribute to the effect of the combination of pembrolizumab and 5-azacitidine on the recruitment of CD8^+^ T cells in CRC. Elucidating the effect of combination of DNMTi and ICI for the activation of immune cells in the TME and tumor regression in pMMR/MSS CRC will be our next lines of investigation.

It has been widely accepted that the treatment with ICIs results in improved survival in patients with dMMR/MSI tumors due to the high tumor mutation burden and neoantigen load, leading to immune cell infiltration, including CD8^+^ T cells, in the TME [13,14]. As we previously reported, the expression of cGAS-STING in tumor cells was maintained at a high level in dMMR/MSI-H CRC, compared to pMMR/MSS CRC, due to less frequent methylation in the promoter region of STING especially. The high expression of tumor cell-intrinsic cGAS-STING might contribute to the activation of immune cells in the TME, resulting in better prognosis for ICI treatment in dMMR/MSI-H CRC. In contrast, in most cases of pMMR/MSS CRC, we revealed that the expression of cGAS-STING in tumor cells was maintained at a low level, at least partly due to epigenetic alterations such as histone methylation in the promoter region of STING, resulting in poor infiltration of CD8^+^ T cells and a worse prognosis in patients with pMMR/MSS CRC.

## 5. Conclusions

A minority of patients (less than 10%) with pMMR/MSS exhibited cGAS^+^/STING^+^ expression with high infiltration of CD8^+^ T cells and thus may potentially benefit from immunotherapy with ICI. Moreover, the expression of cGAS-STING in tumor cells could serve as a potential biomarker for predicting the efficacy of ICI treatment in patients with pMMR/MSS CRC.

## Figures and Tables

**Table 1:** Clinico-pathological characteristics of patients with pMMR CRC.

		pMMR CRC All	cGAS^+^/STING^+^	cGAS^+^/STING^−^	cGAS^−^/STING^+^	cGAS^−^/STING^−^	
		(*n* = 243)	(*n* = 24)	(*n* = 34)	(*n* = 47)	(*n* = 138)	*p*-Value
Age	<70	121	11	20	17	73	0.1523
	70≤	122	13	14	30	65	
Gender	Male	154	18	25	30	81	0.2393
	Female	89	6	9	17	57	
Tumor location	Proximal	78	12	16	16	34	0.0379
	Distal	68	4	10	15	39	
	Rectum	97	8	8	16	65	
Tumor differentiation	Well/Moderate	234	22	31	47	134	0.1116
	Poor	9	2	3	0	4	
T stage	Tis	13	3	0	6	4	0.0113
	T1	31	3	4	6	18	
	T2	35	1	5	14	15	
	T3	101	10	16	13	62	
	T4	63	7	9	8	39	
N stage	N0	144	13	17	31	83	0.4967
	N1-3	99	11	17	16	55	
M stage	M0	213	20	30	44	119	0.5267
	M1	30	4	4	3	19	
TNM stage	0	13	3	0	6	4	0.0658
	I	52	4	7	15	26	
	II	72	6	9	9	48	
	III	76	7	14	14	41	
	IV	30	4	4	3	19	
PD-L1	Positive	12	2	3	2	5	0.5246
	Negative	231	22	31	45	133	
Recurrence	Yes	40	2	5	8	25	0.6752
	No	180	19	26	37	98	
	Not available	23	3	3	2	15	-

pMMR: mismatch repair proficient; CRC: colorectal cancer; cGAS: cyclic GMP-AMP synthase; STING: stimulator of interferon genes; PD-L1: programmed death-ligand 1. Statistical significance within each category of clinical characteristics was determined by Chi-square test comparing the four groups (cGAS^+^/STING^+^, cGAS^+^/STING^−^, cGAS^−^/STING^+,^ and cGAS^−^/STING^−^).

## Data Availability

The datasets generated during and/or analyzed during the current study are available from the corresponding author on reasonable request.

## Supplementary Materials

The following supporting information can be downloaded at: https://www.mdpi.com/article/10.3390/cancers15102826/s1, Figure S1: The evaluation of the tumor cell-intrinsic expression of cyclic GMP-AMP synthase (cGAS) and stimulator of interferon genes (STING) in mismatch repair proficient (pMMR) colorectal cancer (CRC) by immunohistochemistry (IHC); Figure S2: Immunohistochemistry of cGAS and STING in pMMR colorectal cancer CRC; Figure S3: The expression pattern of tumor cell-intrinsic cGAS-STING in mismatch repair deficient (dMMR) CRC; Figure S4: Correlation between mRNA expression of cGAS and STING in pMMR/microsatellite stable (MSS) CRC; Figure S5: Associations between the expression of cGAS-STING in tumor cells and clinico-pathological characteristics of patients with pMMR CRC; Figure S6: Associations between the expression of cGAS-STING in tumor cells and TNM stage of patients with pMMR CRC; Figure S7: Correlation of the tumor cell-intrinsic expression of cGAS-STING and the infiltration of Treg cells in pMMR CRC; Figure S8: Associations between tumor cell-intrinsic expression of cGAS-STING and patient survival with pMMR CRC; Figure S9: Associations between tumor cell-intrinsic expression of cGAS-STING and patient survival with pMMR CRC in each stage; Table S1: Clinico-pathological characteristics of patients with dMMR CRC.
