# Supplementary material for: The Impact of Tumor Cell-Intrinsic Expression of Cyclic GMP-AMP Synthase (cGAS)-Stimulator of Interferon Genes (STING) on the Infiltration of CD8+ T Cells and Clinical Outcomes in Mismatch Repair Proficient/Microsatellite Stable Colorectal Cancer
Source: Cancers (Basel). 2023 May 18;15(10):2826. doi: 10.3390/cancers15102826 (PMC10216055; doi:10.3390/cancers15102826)
Supplement: Supplementary file 1 [file cancers-15-02826-s001.zip › cancers-2284600-supplementary.pdf]

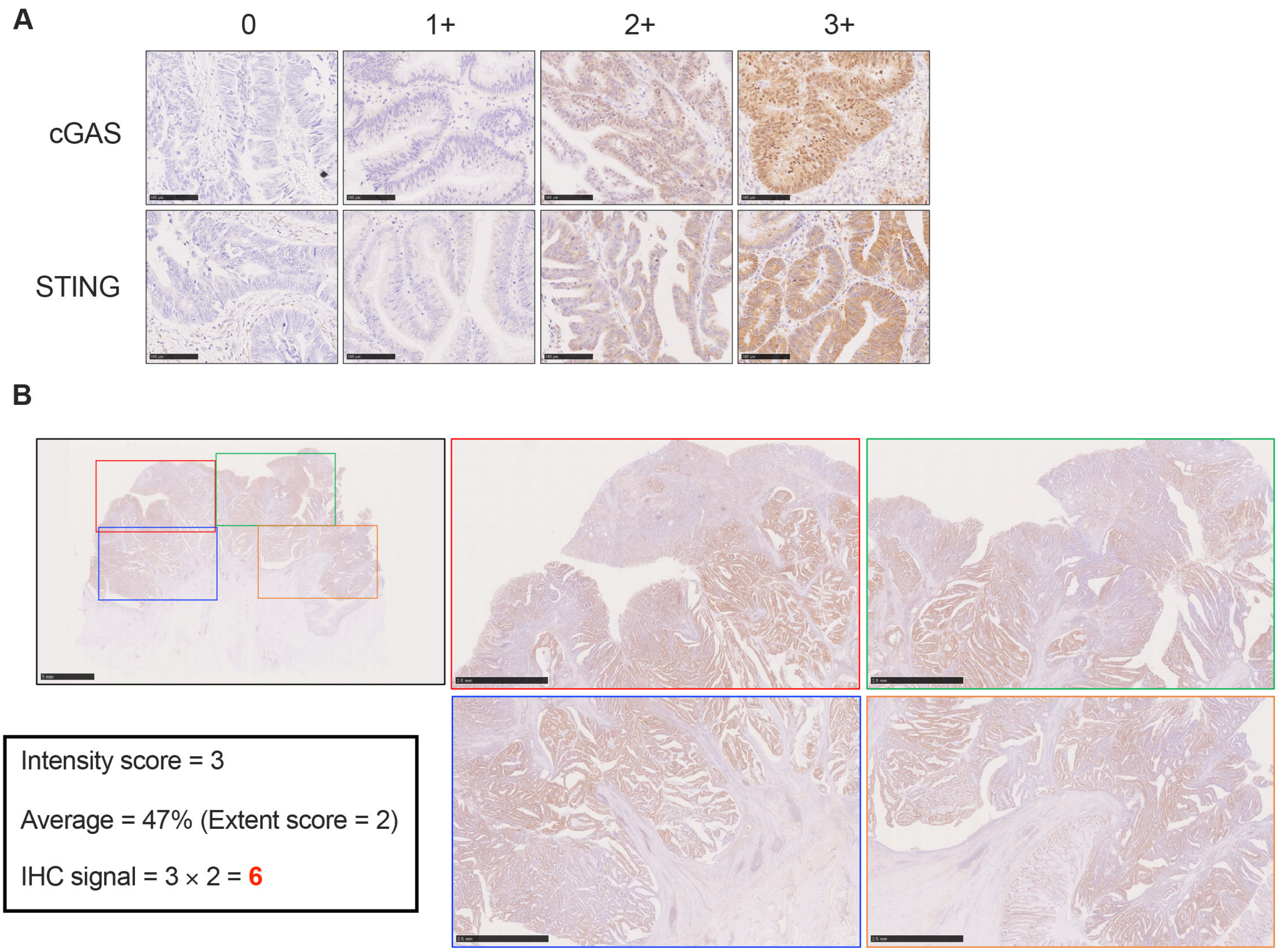

Supplementary Figure S1. The evaluation of the tumor cell-intrinsic expression of cyclic GMP-AMP synthase (cGAS) and stimulator of interferon genes (STING) in mismatch repair proficient (pMMR) colorectal cancer (CRC) by Immunohistochemistry (IHC). (**A**) Representative IHC staining images for cGAS (**upper**) and STING (**lower**) in surgically resected CRC specimens. The intensity score was graded by staining in the cytoplasm as follows: 0 (none), 1+ (weak), 2+ (moderate), or 3+ (strong). Scale bars, 100 μm. (**B**) The scanned whole tissue image was partitioned into multiple regions (red, blue, green, and orange) and the proportion of stained cytoplasm was determined by calculating the mean value of each region (red, blue, green, and orange). The extent score was estimated on the basis of the percentage of the area of stained cytoplasm (0 for no staining at all, 1 for <10%, 2 for 10% to 50%, and 3 for >50% of tumor cells stained). The final score was determined by multiplying the extent score and intensity score (IHC signal, 0–9), and IHC signal ≧3 was considered as positive expression. Scale bars, 2.5 mm or 5 mm.

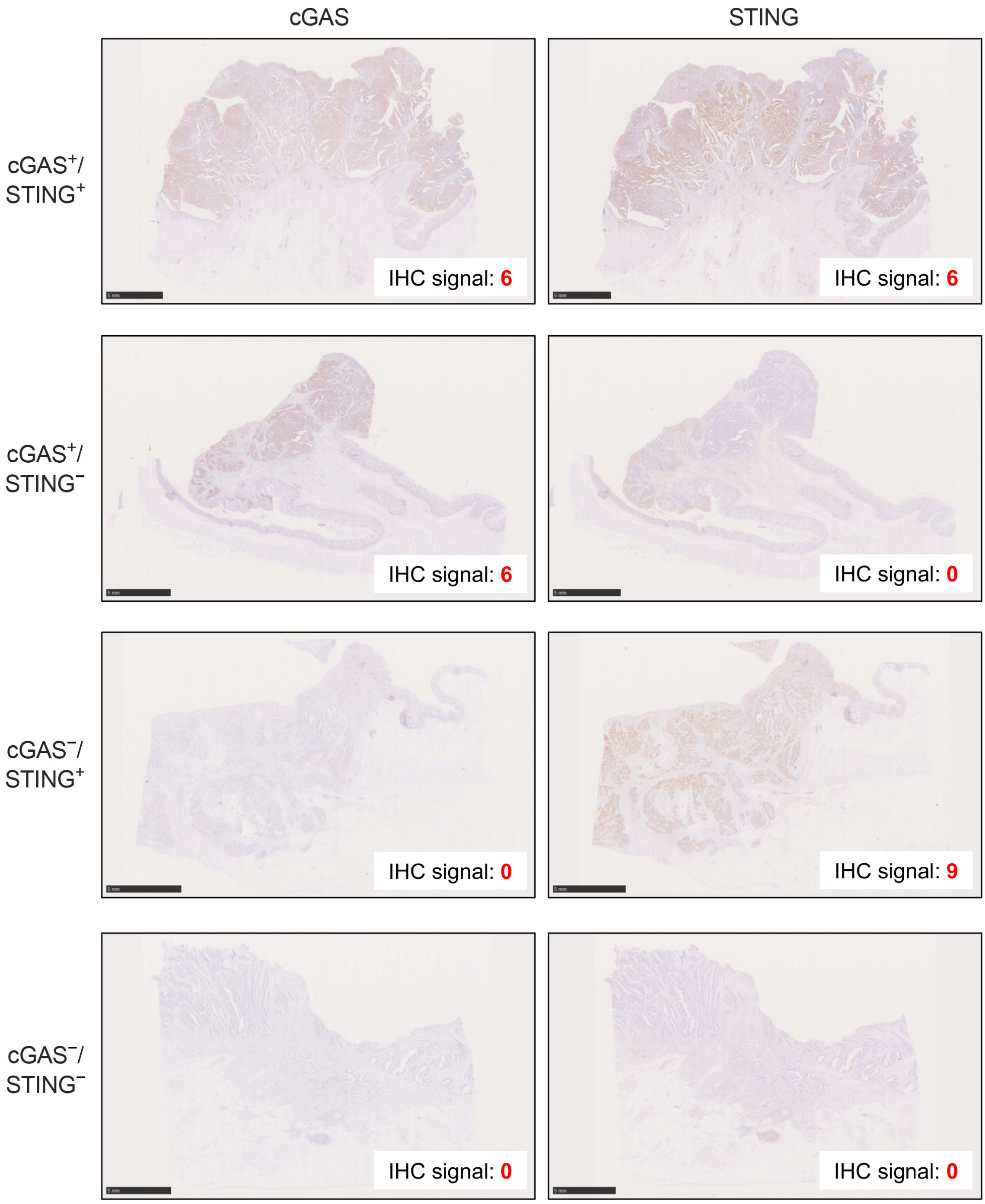

Supplementary Figure S2. IHC of cGAS and STING in pMMR CRC. Representative IHC staining images of whole tissue sections for cGAS (**left**) and STING (**right**). Enlaraged regions were shown in Figure 1C. IHC signals are indicated in each image. Scale bars, 5 mm.

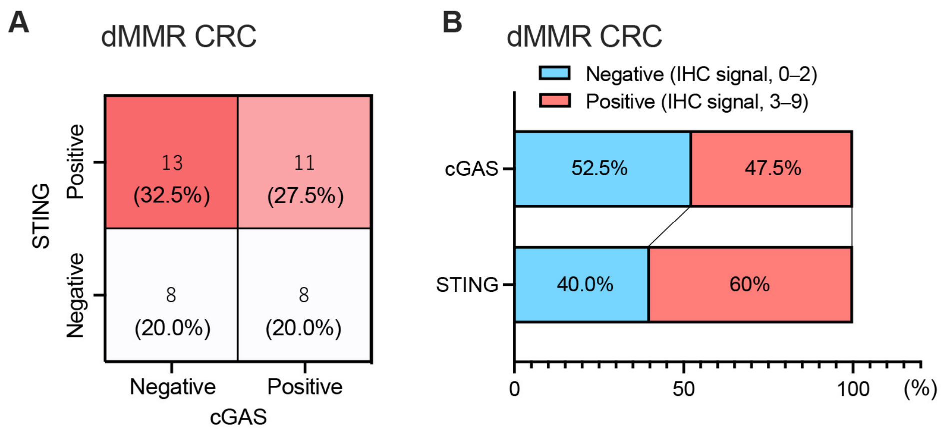

Supplementary Figure S3. The expression pattern of tumor cell-intrinsic cGAS-STING in mismatch repair deficient (dMMR) CRC. (**A**) Proportions of $cGAS^+/STING^+$, $cGAS^+/STING^-$, $cGAS^-/STING^+$, and $cGAS^-/STING^-$ dMMR CRCs. The intensity of the heatmap represents number of each proportion, with values ranging from 8 to 13 shown as white and red. (**B**) The percentage of positive or negative cases for the expression of cGAS or STING in tumor cells in dMMR CRC (FMU cohort). cGAS- or STING-positive cases are highlighted in red and cGAS- or STING-negative are highlighted in blue. The percentage of cGAS-STING positivity is indicated in the graph.

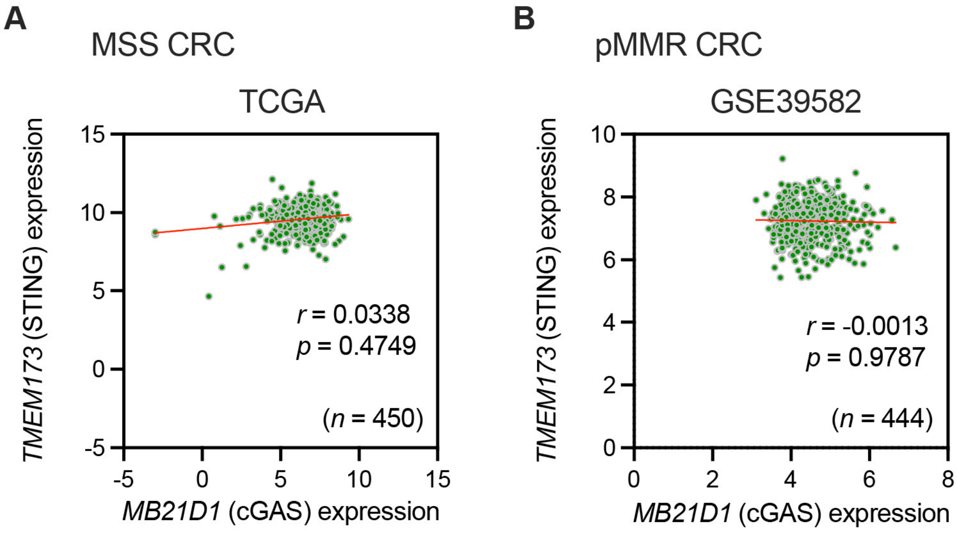

Supplementary Figure S4. Correlation between mRNA expression of cGAS and STING in pMMR/microsatellite stable (MSS) CRC. (**A and B**) Correlations between mRNA expression of *MB21D1* (cGAS) and *TMEM173* (STING) in pMMR/MSS CRC. Gene expression datasets were obtained from TCGA (**A**) or GSE39582 (**B**). Statistical significance was determined by the Spearman correlation test (**A**,**B**).

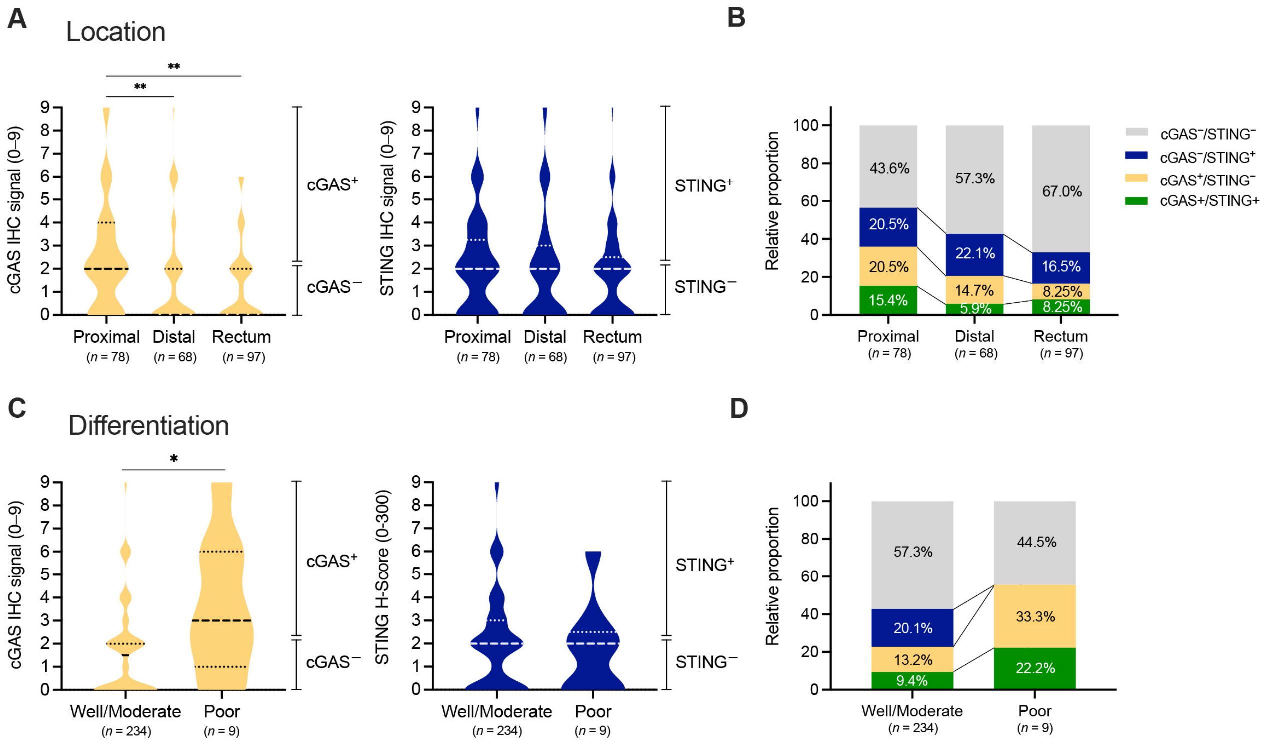

Supplementary Figure S5. Associations between the expressoin of cGAS-STING in tumor cells and clinico-pathological characteristics of patients with pMMR CRC. (**A and C**) Correlations between IHC signals of cGAS (**left**) or STING (**right**) and tumor locations (**A**) or histrogical differentiations (**C**) in patients with pMMR CRC (FMU cohort). Lines in the right side of the graphs indicate the ranges of cGAS-STING-positive or cGAS-STING-negative. (**B and D**) Correlations between the percentages of cGAS+/STING+ (green), cGAS+/STING− (yellow), cGAS−/STING+ (blue), or cGAS−/STING− (grey) tumors and tumor locations (**B**) or histrogical differentiations (**D**) in patients with pMMR CRC (FMU cohort). Medians and quartiles are shown in violin plots. *p <0.05, **p <0.01. Statistical significance was determined by the Kruskal-Wallis test with post hoc Dunn's multiple comparisons test (**A**) or Mann-Whitney test (**C**).

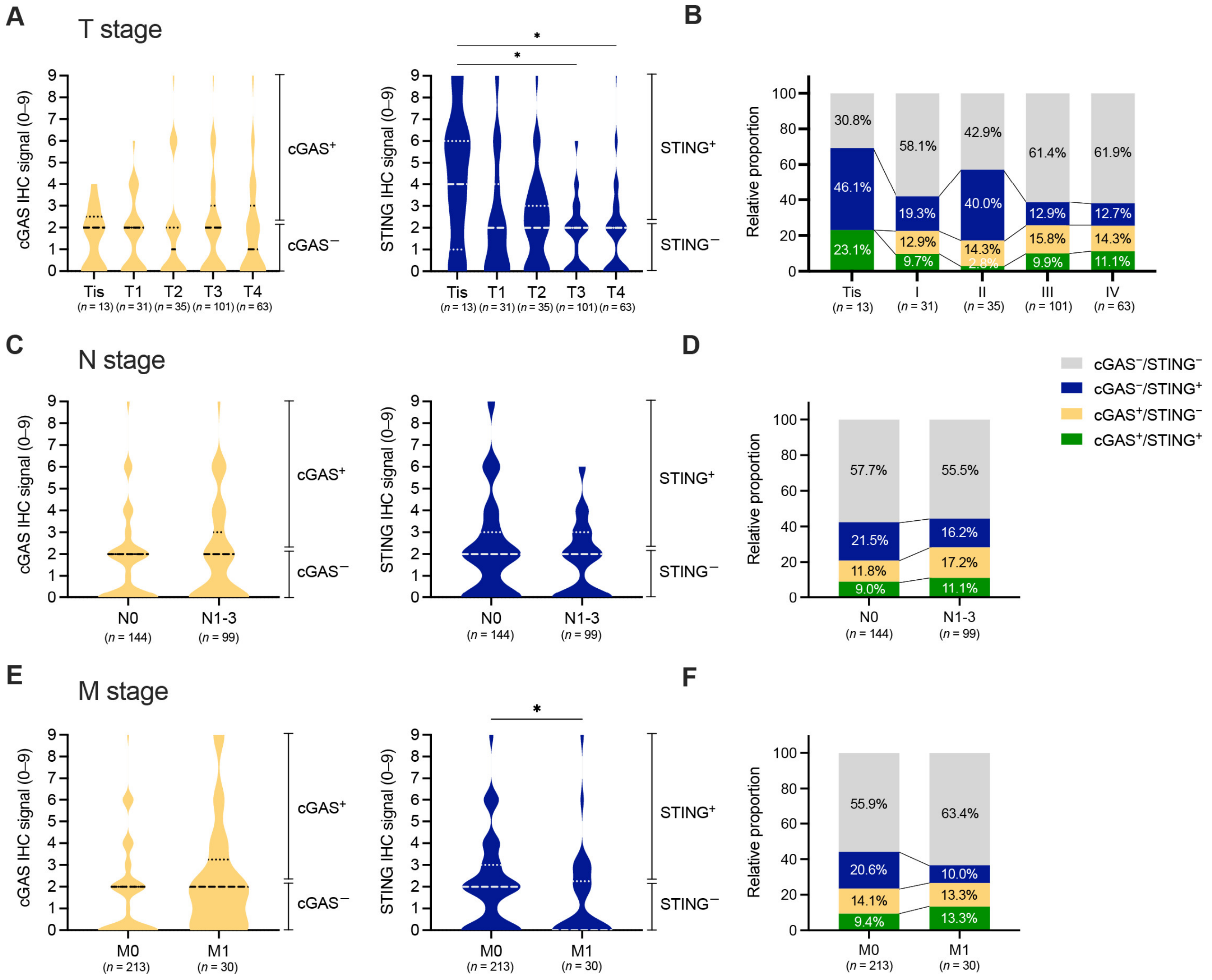

Supplementary Figure S6. Associations between the expressoin of cGAS-STING in tumor cells and TNM stage of patients with pMMR CRC. (**A, C, and E**) Correlations between IHC signals of cGAS (**left**) or STING (**right**) and T stage (**A**), N stage (**C**), or M stage (**E**) in patients with pMMR CRC (FMU cohort). Lines in the right side of the graphs indicate the ranges of cGAS-STING-positive or cGAS-STING-negative. (**B, D and F**) Correlations between the percentages of cGAS$^+$/STING$^+$ (green), cGAS$^+$/STING$^-$ (yellow), cGAS$^-$/STING$^+$ (blue), or cGAS$^-$/STING$^-$ (grey) tumors and T stage (**B**), N stage (**D**), or M stage (**F**) in patients with pMMR CRC (FMU cohort). Medians and quartiles are shown in violin plots. *p <0.05. Statistical significance was determined by the Kruskal-Wallis test with post hoc Dunn's multiple comparisons test (**A**) or Mann-Whitney test (**C**,**E**).

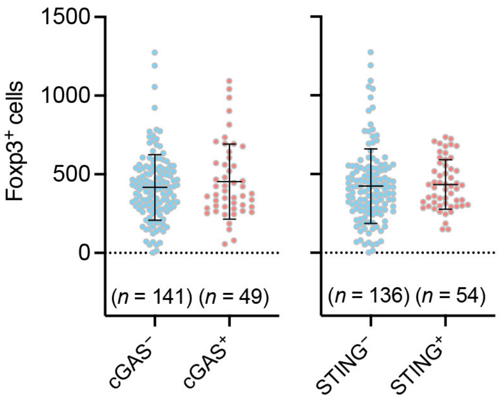

Supplementary Figure S7. Correlation of the tumor cell-intrinsic expression of cGAS-STING and the infiltration of Treg cells in pMMR CRC. The number of tumor-infiltrating Treg cells (Foxp3⁺ cells) between cGAS⁺ (red) and cGAS⁻ (blue) (**left**), or STING⁺ (red) and STING⁻ (blue) (**right**) pMMR CRCs (FMU cohort). Means ± SD are shown in dot plots. Statistical significance was determined by the Unpaired t test (**left**) or Mann-Whitney test (**right**).

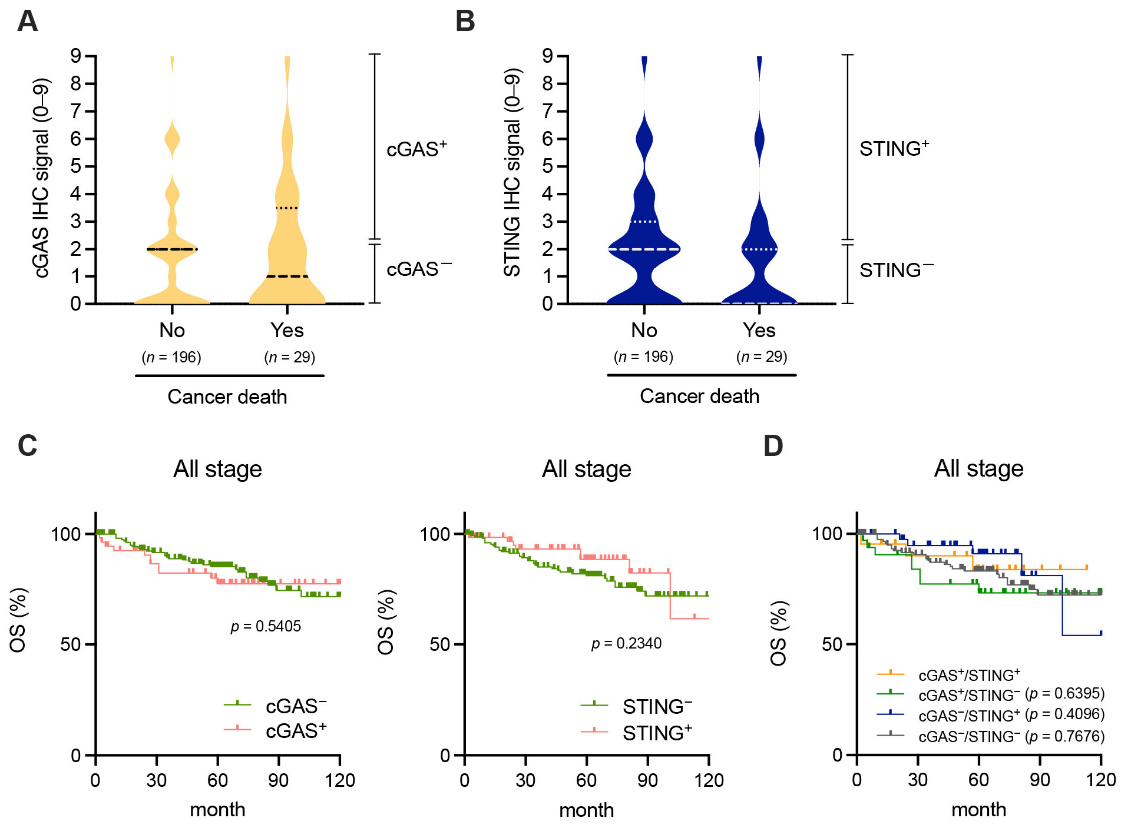

Supplementary Figure S8. Associations between tumor cell-intrinsic expression of cGAS-STING and patient survival with pMMR CRC. (**A and B**) Correlations between IHC signals of cGAS (**A**) or STING (**B**) and 10-year survival in patients with pMMR CRC (FMU cohort). Lines in the right side of the graphs indicate the ranges of cGAS-STING-positive or cGAS-STING-negative. (**C**) Kaplan-Meier curves for 10-year overall survival (OS) in patients with cGAS+ (red) or cGAS− (green) (**left**), and STING+ (red) or STING− (green) (**right**) pMMR CRCs (FMU cohort). (**D**) Kaplan-Meier curves for 10-year OS in patients with cGAS+/STING+ (yellow), cGAS+/STING− (green), cGAS−/STING+ (blue), and cGAS−/STING− (grey) pMMR CRCs (FMU cohort). *p*-values vs. cGAS+/STING+ pMMR CRC are shown in the graph. Medians and quartiles are shown in violin plots. Statistical significance was determined by the Mann-Whitney test (**A**,**B**) or Log-rank test (**C**,**D**).

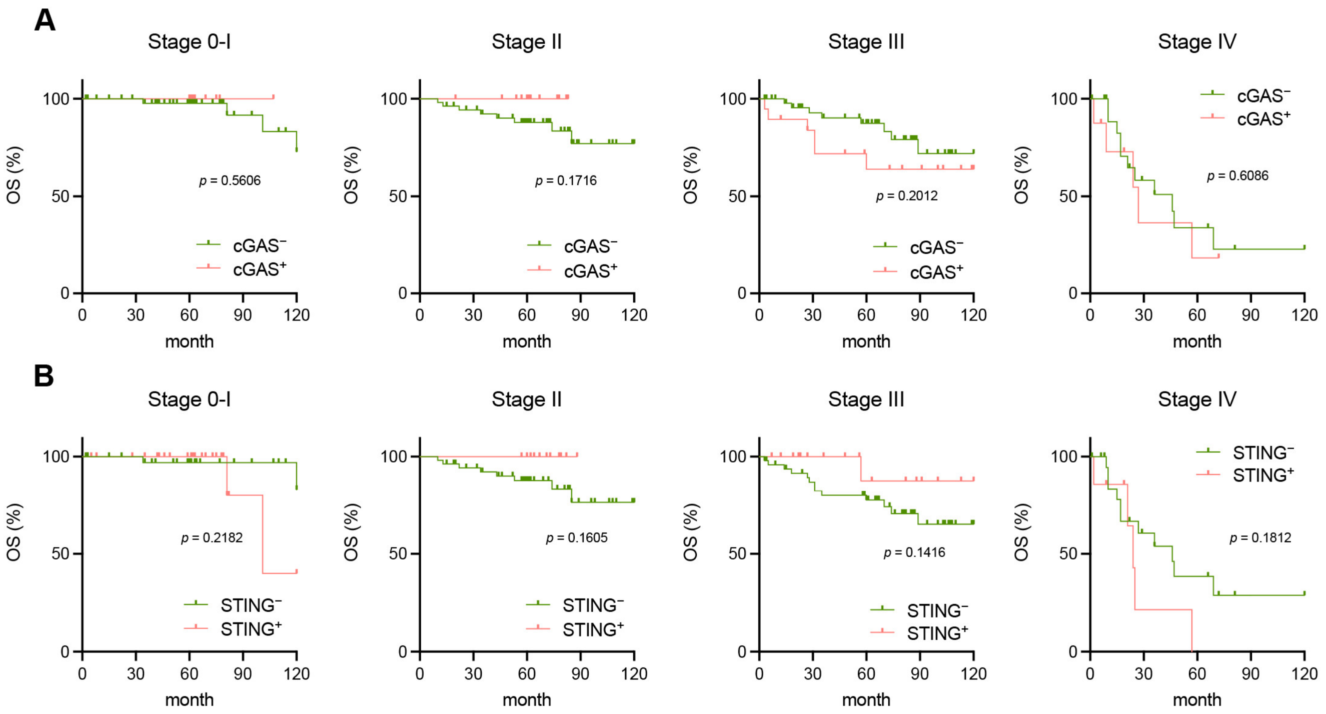

Supplementary Figure S9. Associations between tumor cell-intrinsic expression of cGAS-STING and patient survival with pMMR CRC in each stage. (**A and B**) Kaplan-Meier curves for 10-year OS in patients with $cGAS^{+}$ (red) or $cGAS^{-}$ (green) (**A**), and $STING^{+}$ (red) or $STING^{-}$ (green) (**B**) pMMR CRCs in each stage (FMU cohort). Statistical significance was determined by the Log-rank test (**A**,**B**).

Supplementary Table S1. Clinico-pathological characteristics of patients with dMMR CRC.

		dMMR CRC All	$cGAS^{+}$/ $STING^{+}$	$cGAS^{+}$/ $STING^{-}$	$cGAS^{-}$/ $STING^{+}$	$cGAS^{-}$/ $STING^{-}$	
		**(*n* = 40)**	**(*n* = 11)**	**(*n* = 8)**	**(*n* = 13)**	**(*n* = 8)**	***p*-Value**
Age	<70	20	4	2	11	3	0.0228
	70≤	20	7	6	2	5	
Gender	Male	19	5	3	7	4	0.9034
	Female	21	6	5	6	4	
Tumor location	Proximal	29	10	7	9	3	0.1908
	Distal	6	0	1	2	3	
	Rectum	5	1	0	2	2	
Tumor differentiation	Well/Moderate	28	8	6	10	4	0.5819
	Poor	12	3	2	3	4	
T stage	Tis	0	0	0	0	0	NA
	T1	1	1	0	0	0	
	T2	9	5	1	2	1	
	T3	20	4	3	7	6	
	T4	10	1	4	4	1	
N stage	N0	27	8	5	8	6	0.6852
	N1-3	12	3	3	5	1	
	Not available	1	0	0	0	1	-
M stage	M0	38	10	8	12	8	0.699
	M1	2	1	0	1	0	
TNM stage	0	0	0	0	0	0	NA
	I	9	5	1	2	1	
	II	18	3	4	6	5	
	III	10	2	3	4	1	
	IV	3	1	0	1	1	
PD-L1	Positive	15	5	3	6	1	0.416
	Negative	25	6	5	7	7	
Recurrence	Yes	2	0	1	0	1	0.3343
	No	37	11	7	13	6	
	Not available	1	0	0	0	1	-

dMMR; mismatch repair deficient, CRC; colorectal cancer, cGAS; cyclic GMP-AMP synthase, STING; stimulator of interferon genes, PD-L1; programmed death-ligand 1. Statistics significance within each category of clinical characteristics was determined by Chi-square test among the four groups ($cGAS^{+}$/$STING^{+}$, $cGAS^{+}$/$STING^{-}$, $cGAS^{-}$/$STING^{+}$, and $cGAS^{-}$/$STING^{-}$).
